# Prevalence and patterns of traditional and e-cigarette use, and factors associated with e-cigarette use

**DOI:** 10.3389/fpubh.2025.1698254

**Published:** 2025-12-18

**Authors:** Rifah Anwar Assadi, Noorah Ali Omar Alshimmari, Jayadevan Sreedharan

**Affiliations:** 1 College of Medicine, Gulf Medical University, Ajman, United Arab Emirates; 2 LAIQ, Sheikh Khalifa Medical City Ajman, Ajman, United Arab Emirates; 3 Department of Community Medicine, College of Medicine, Gulf Medical University, Ajman, United Arab Emirates

**Keywords:** vaping, e-cigarette, tobacco use, smoking, UAE, behavioral factors, nicotine products, current smokers

## Abstract

**Background:**

The use of electronic cigarettes (e-cigarettes) is rising globally, including in the UAE. They are often marketed as harm reduction tools and potential aids for smoking cessation, but their long-term health effects remain unclear.

**Objective:**

To determine the prevalence and patterns of e-cigarette and tobacco use among adults in the UAE, and to assess sociodemographic and behavioral factors influencing e-cigarette use.

**Methods:**

A cross-sectional study was conducted in 2024 using a structured, self-administered questionnaire. Participants aged 18 years and older were recruited through convenience sampling across public places in the UAE. The questionnaire, available in English, Arabic, Urdu, and Hindi, included items on demographics, smoking and vaping behavior, quit attempts, and influencing factors. Data were analyzed using SPSS version 29. Chi-square tests were used for bivariate analysis to identify associations (*p* ≤ 0.05). Multivariable logistic regression was performed to determine independent predictors of e-cigarette use, with adjusted odds ratios (AOR) and 95% confidence intervals reported.

**Results:**

Among the 1,500 participants, 27.6% reported ever using tobacco products, and 14.1% were current smokers. Cigarettes were the most common product used (22.3%), followed by e-cigarettes (6.1%), shisha (5.6%), and dhokha (1.4%). E-cigarette use was more prevalent among younger adults (10.1% among 18–24-year-olds), females (7.8%), those with higher education, singles, and students. Behavioral factors such as stress, curiosity, social influence, and coping mechanisms were significantly associated with e-cigarette use.

**Conclusion:**

E-cigarette use in the UAE is influenced by a combination of sociodemographic and behavioral factors. The findings highlight the importance of separating e-cigarette use from traditional tobacco consumption in public health research and interventions, particularly when targeting younger populations.

## Introduction

Tobacco use remains a leading global risk factor for disability-adjusted years of life lost (1). It is a major cause of death from cardiovascular disease, cancer, and pulmonary disease (2). Additionally, it is a notable risk factor for reproductive disorders, osteoporosis, and fire-trauma-related injuries (2). Globally, smoking tobacco contributed to 7.69 million fatalities in 2019 (1). Despite significant advancements in global tobacco control, the tobacco epidemic still poses a substantial public health issue. The 2025 WHO Tobacco Trends Report indicates that around 1.2 billion individuals worldwide continue to use tobacco, with a 27% decrease in prevalence since 2010, yet it still impacts one in five adults. Tobacco consumption remains responsible for millions of preventable deaths each year. The report also points out the swift increase in e-cigarette usage, with more than 100 million users globally—including 86 million adults and 15 million adolescents—raising alarms about nicotine addiction in young people. (3).

Tobacco smoke contains nicotine, water, alkaloids, and tar, suspended in gas. Several thousand chemicals, free radicals, oxidizing chemicals, and particulates contribute to disease (2). Nicotine is the addictive in tobacco products, activating reward circuits as seen with drugs cocaine and heroin (4).

More than 80% of users are cigarette smokers, but the availability of a diverse range of non-combustible products and combustible products may change the landscape of consumption (4). Since 2007, electronic nicotine delivery systems (ENDS), also called e-cigarettes or vaping products, have been introduced (5). These products contain a battery which powers a heating element to aerosolize liquid containing nicotine, humectants and flavorings (6). Importantly, e-cigarettes do not contain tobacco but may contain nicotine.

There has been a notable decline in cigarette smoking in high-income countries, but this does not eliminate the need for tobacco control. Electronic nicotine delivery systems are primarily marketed in high-income countries, while traditional cigarettes remain prevalent in lower middle-income countries (7). Electronic cigarettes (e-cigarettes) comprise of ENDS (Electronic nicotine delivery system) and ENNDS (Electronic non-nicotine delivery systems) with others like e-cigar and e-pipe. Vaping is colloquially referred to as use of END or ENNDS (8). While the long-term health effects of vaping are not fully understood, current evidence suggests that e-cigarettes are not without risk (8).

The UAE population encompasses a large proportion of culturally diverse migrants. High levels of cigarette smoking and smokeless tobacco use are observed among migrants from Pakistan, India, and Bangladesh. Loss of productivity is greatly associated with cigarette smoking (9). UAE university students study revealed that 15.1% smoked conventional cigarettes or e-cigarettes, of which 4% accounted for e-cigarettes (10). According to a recent study, e-cigarette use is prevalent among university students in the UAE, with around 23% of students having used an e-cigarette in the past month, 37% used e-cigarette in their lifetime (11). A national health survey conducted among adults in the UAE 2017–2018 found that the overall prevalence of current smoking was 9.1% among the selected population (12).

E-cigarettes have shown potential as smoking cessation aids in clinical trials, but their safety and efficacy require further evaluation (7, 13–15). Like conventional cigarettes, e-cigarettes attract users by mimicking the look and taste of cigarettes and allowing individuals to continue the hand-to-mouth ritual of smoking (16).

Every country has its own regulations regarding e-cigarettes, and these regulations are constantly evolving (17). The World Health Organization (WHO) has stated that ENDS require regulation to prevent promotion to non-smokers and youth (18–20). In UAE, the sale of tobacco products is forbidden to under 18 years according to federal law (21). There is a lack of detailed surveillance data on the burden of various patterns of smoking, e-cigarette/ENDS use, and smoking cessation. This study aimed to assess the prevalence and determinants of e-cigarette and traditional tobacco use among adults in the United Arab Emirates (UAE).

## Methods

This study utilized a cross-sectional design. Eligible participants were individuals aged 18 years and above, from any gender or nationality, who consented to take part in the study. Those who declined participation were excluded.

The sample size was determined based on previous research indicating a 23% prevalence of e-cigarette use among young adults in the UAE (11). After adjusting for a 10% nonresponse rate, the final target sample size was approximately 1,473, rounded up to 1,500 participants, recruited through convenience sampling. Recruitment sites—such as universities, health centers, and workplaces—were chosen for their logistical feasibility and accessibility, as they represent public venues with high adult foot traffic. Most participants were recruited from Ajman. While this approach facilitated the inclusion of a diverse group of adults within a limited timeframe, it inherently carries the risk of selection bias and overrepresentation of Ajman residents and blue-collar migrant workers, reflecting the emirate’s demographic profile. Consequently, the findings may either overestimate or underestimate the true prevalence of smoking and vaping behaviors at the national level.

Data collection occurred between 9 February 2024 and 9 May 2024, using a self-administered questionnaire. The survey instrument captured information on sociodemographic attributes, patterns of tobacco and e-cigarette use, cessation attempts, and factors influencing initiation and continuation of use. The questionnaire underwent both face and content validation by three public health experts and was piloted prior to the main study to ensure clarity and relevance. To accommodate the UAE’s diverse population, the tool was translated and back-translated into Hindi, Urdu, and Arabic. The final English version of the questionnaire is provided as Supplementary File S1.

Ethical approval was secured from the Institutional Review Board of Gulf Medical University (IRB-COM-STD-70-DEC-2023) and the Research Ethics Committee of the Ministry of Health and Prevention, UAE (MOHAP/DXB-REC/D.D-J/N0.162/2023). Written informed consent was obtained from all participants. Participation was entirely voluntary and anonymous, with individuals free to withdraw at any stage. Data were collected both online and in person, depending on participant preference and feasibility. For online respondents, consent was digitally documented by the participant’s explicit selection of “Yes” or “No,” with oversight from the data collection team to ensure procedural integrity. The operational definitions used in this study are listed in Table 1 to ensure consistency in data analysis.

**Table 1:** Operational definitions.

Term	Conceptual definition	Operational definition
Novice/experimental smokers	An individual who is in the initial stages of experimenting with and developing a smoking habit (42).	Individual who has smoked, but have not yet reached the 100-cigarette/20 shisha or vape or dokha sessions in lifetime threshold to be considered as established smokers, regardless of their current smoking status
Never smoker	An adult who has never smoked, or who has smoked less than 100 cigarettes in his or her lifetime (22).	An individual who has never smoked.
Current smoker	An adult who has smoked 100 cigarettes in his or her lifetime and who currently smokes cigarettes. Beginning in 1991 this group was divided into “everyday” smokers or “somedays” smokers (22).	If participant has reported having smoked at least 100 cigarettes/20 shisha or vape or dhokha sessions in their lifetime and currently engages in smoking since past 12 months.
Former smoker	An adult who has smoked at least 100 cigarettes in his or her lifetime but who had quit smoking at the time of interview (study) (22).	A participant who has disclosed, in their questionnaire responses, a history of having smoked at least 100 cigarettes/20 shisha or vape or dhokha sessions in their lifetime but states that they have quit smoking at the time.
Occasional smoker	An adult who has smoked at least 100 cigarettes in his or her lifetime, who smokes now, but does not smoke every day (22).	Participants who have smoked atleast 100 cigarettes/20 shisha or vape or dokha sessions in their lifetime but do not currently smoke daily and may not have smoked in the past 12 months.

The 100-cigarette threshold is a standard classification used by CDC (22) to define established smoking behavior. For e-cigarettes and shisha, an equivalent threshold of ≥20 sessions were used to approximate habitual exposure. This cutoff is consistent with previous related study (23) and intended to differentiate experimental from established use rather than quantify exact nicotine exposure. Frequency, duration, and nicotine concentration were not directly measured.

All collected data will be securely archived for a period of 3 years. Statistical analysis was performed using SPSS version 29. Descriptive statistics (frequencies and percentages) and inferential statistics were employed, with statistical significance set at a *p*-value ≤ 0.05. Bivariate analyses were performed using chi-square tests, ensuring all cells met minimum expected count assumptions where possible; where cell counts were <5 (e.g., gold-collar or small nationality subgroups), results were interpreted cautiously. Multivariable binary logistic regression models were used to estimate adjusted odds ratios (AORs) for e-cigarette use. Sociodemographic covariates entered included age, gender, education, nationality, marital status, religion and employment status. Behavioral variables such as stress, curiosity, family/friends using tobacco, spiritual beliefs, Work/Environment stress, coping strategy, peer influence, and marketing exposure were also tested. We assessed collinearity among the independent variables using the Chi-square test of association. Variables that showed significant associations were excluded from the multiple logistic regression model to avoid multicollinearity.

## Results

### Demographic characteristics of study population

The total number of participants was 1,500. Table 2 provides a comprehensive overview of the demographic and socio-economic characteristics of the study participants. The age distribution reveals that the largest group, comprising 35%, was aged 25–34 years, while 26.9% were in the 18–24 age. Gender representation was predominantly male at 60.5%. Regarding education, a significant portion of participants (37%) held university degrees while 7.1% did not finish primary school. In terms of nationality, the Southeast Asia Region (SEARO) was the most represented, accounting for 40.9%, followed by the Eastern Mediterranean Region (EMRO) at 34.7%. Marital status showed that 54.8% had been married at some point in their lives. The religious composition indicated a majority of Muslims (67.4%). Geographically, most participants resided in Ajman (58.5%), with Dubai (19.3%). Employment status indicated that 62.4% were employed. Work category analysis revealed that blue-collar workers constituted the largest segment at 44.1%, followed by white-collar workers at 31.4%. Finally, income levels highlighted that the most common earnings fell within the 2,000–4,999 AED bracket (30.0%), and only 2.3% reporting earnings of 30,000 AED or more. These findings illuminate the diverse socio-economic landscape of participants in the UAE.

**Table 2:** Frequency distribution of sociodemographic and characteristics of the participants (*N* = 1,500).

Variable	Group	No.	%
Age	18–24	404	26.9
	25–34	525	35.0
	35–44	254	16.9
	45–54	191	12.7
	>55	126	8.4
Gender	Male	908	60.5
	Female	592	39.5
Education	Below University	887	59.1
	Above University	613	40.9
Nationality^a^	AFR	128	8.5
	AMR	32	2.1
	SEAR	614	40.9
	EUR	80	5.3
	EMR	520	34.7
	WPR	126	8.4
Marital status	Never married	678	45.2
	Ever married	822	54.8
Religion	Muslim	1,011	67.4
	Christian	235	15.7
	Others	254	16.9
Residence	Abu Dhabi	32	2.1
	Dubai	289	19.3
	Sharjah	251	16.7
	Ajman	878	58.5
	Umm Al Quwain	27	1.8
	Ras Al Khaimah	8	0.5
	Fujairah	15	1.0
Employment	Employed	936	62.4
	Student	194	12.9
	Self Employed	135	9.0
	Other	235	15.7
Work categories^b^	White collar	308	32.9
	Blue collar	468	50.1
	Green collar	18	1.9
	Gold collar	63	6.7
	Others	78	8.3
Income level (in AED)	Below 2,000	305	29.4
	2,000–4,999	311	30.0
	5,000–9,999	160	15.4
	10,000–14,999	104	10.0
	15,000–19,999	80	7.7
	Above 20,000	78	7.5

^a^AFR, African Region; EMR, Eastern Mediterranean Region; SEAR, Southeast Asia Region; AMR, Region of the Americas; WPR, Western Pacific Region; EUR, European Region.

^b^White-collar jobs refer to professional, managerial, or administrative roles typically performed in office environments. Blue-collar jobs involve manual labor or skilled trades, often in industrial or technical fields. Green-collar jobs are related to environmental sustainability, renewable energy, and conservation efforts. Gold-collar jobs describe highly skilled, specialized professionals in fields like medicine, law, IT, and finance. Other classifications include pink-collar (service and caregiving roles), gray-collar (technical or supervisory roles), black-collar (hazardous or stigmatized work), and red-collar (government or agricultural jobs).

### Prevalence of tobacco smoking

**Figure 1:**
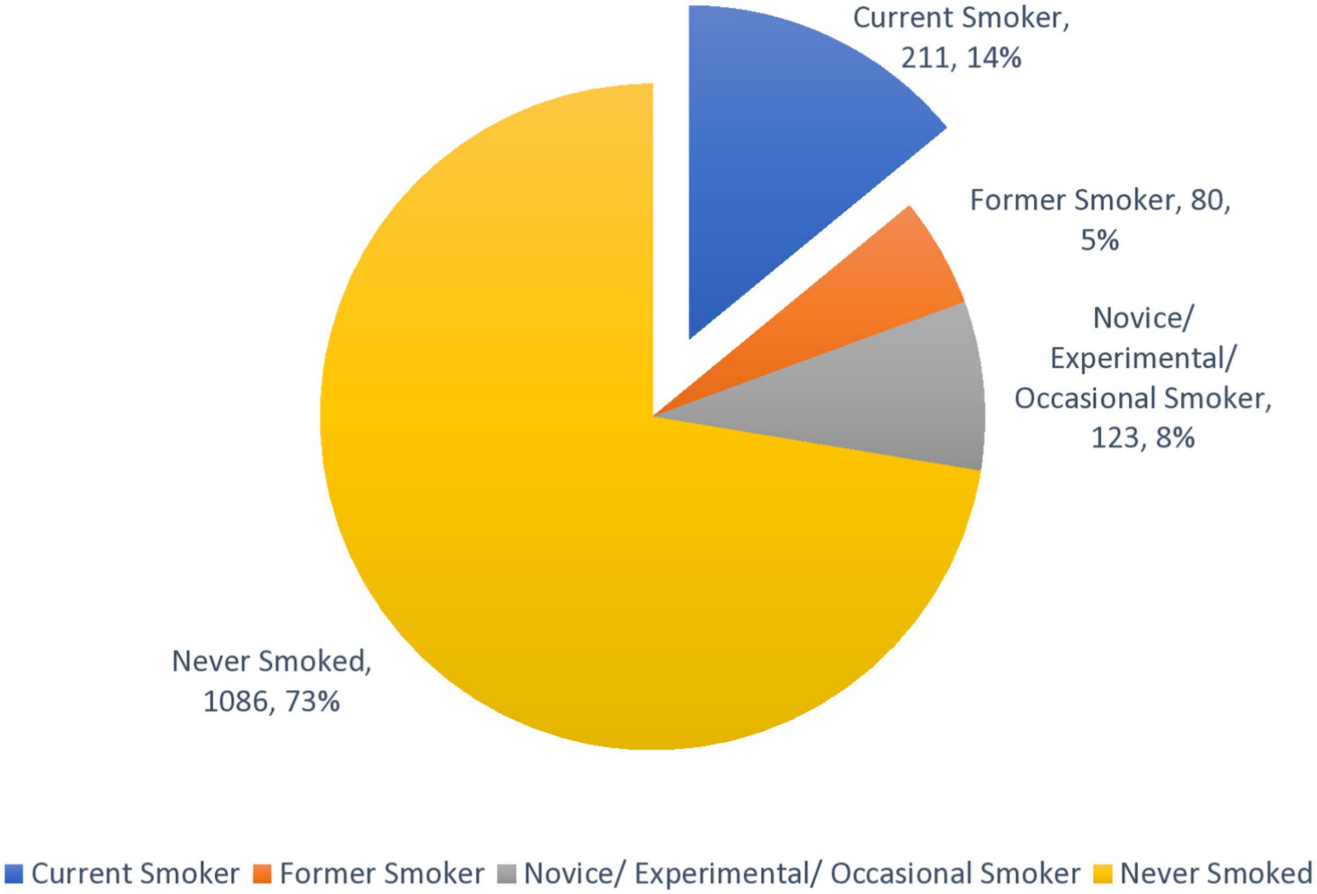
Prevalence of tobacco smoking among participants with distribution of ever smoking (*N* = 1,500).

A significant majority of the population (72.4%) have never smoked. A smaller yet substantial portion (27.6%) have smoked at some point (Figure 1). Among smokers, 51% are current smokers, 19.3% are former smokers. When viewed as a proportion of the total study population, 14.1% were current smokers, 5.3% were former smokers, and 8.2% were novice or occasional smokers (Figure 1).

### Type and pattern of tobacco and e-cigarette use

The most used form of tobacco is cigarettes, with 22.3% of the population using them. Vaping or using e-cigarettes is the second most common, with 6.1% of the population. Shisha is used by 5.6% of the population. A smaller portion of the population, 1.4%, uses Dhokha or Midwakh. Only 0.5% of the population uses other tobacco products. Single users of tobacco products are more prevalent than poly users, with 78.7% of respondents falling into the single user category.

**Figure 2:**
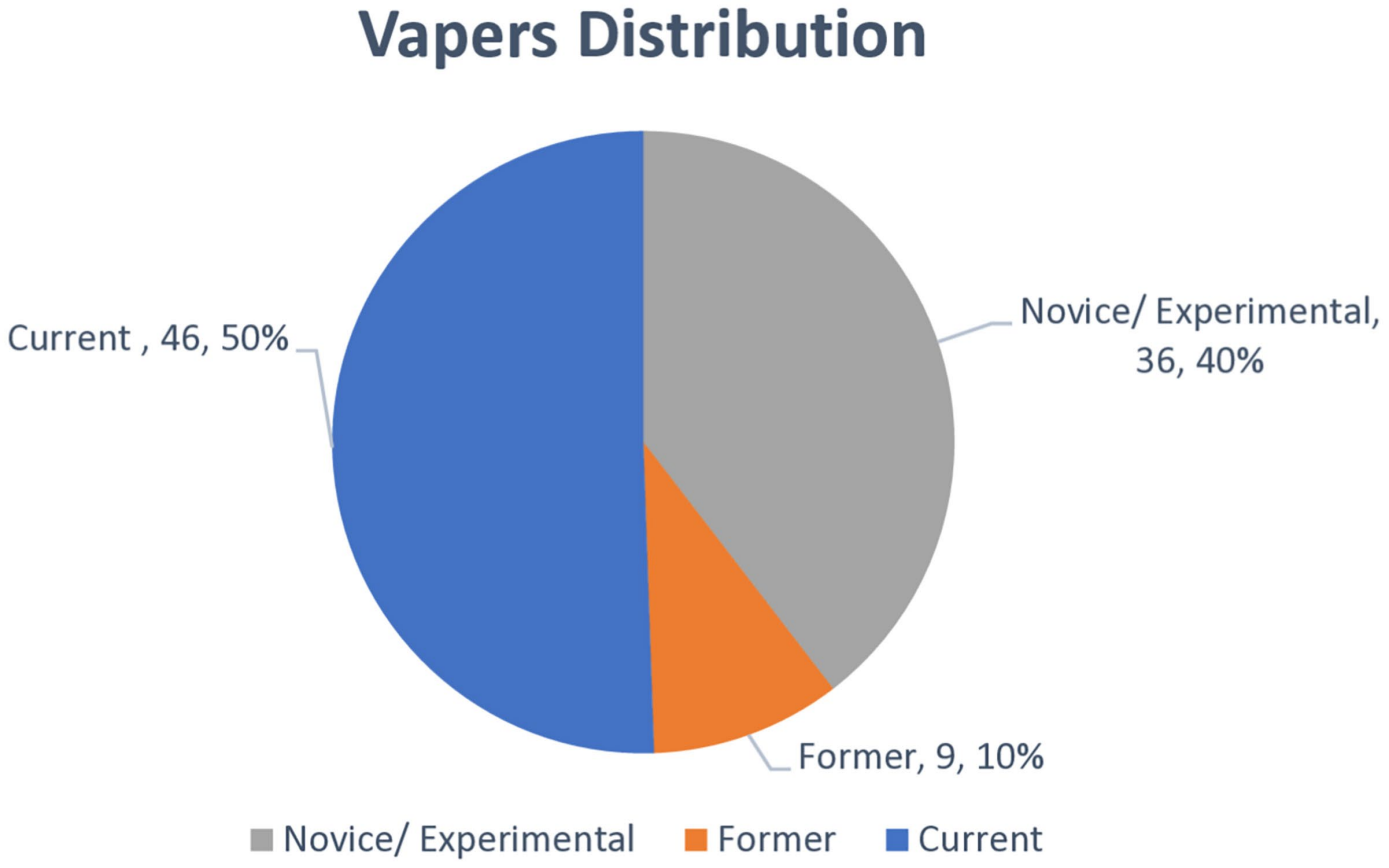
Distribution of novice, current, former users among vapers (*N* = 91).

Figure 2 illustrates the distribution of e-cigarette usage patterns among participants who reported ever using e-cigarettes (*N* = 91). The largest proportion were current vapers, comprising 3.1% (*n* = 46) of the total study population. Novice, experimental, or occasional users accounted for 2.4% (*n* = 36), reflecting individuals who have tried vaping but do not use it regularly. A smaller group, 0.6% (*n* = 9), were identified as former vapers—individuals who previously used e-cigarettes but had since stopped.

### Factors associated with vaping

Table 3 examines the association between sociodemographic characteristics and vaping behavior, revealing several significant findings. Age is notably associated with vaping, with younger individuals (18–24 years) exhibiting a higher prevalence (10.1%). Gender differences are evident, with males vaping at a rate of 5% compared to 7.8% for females. Education also shows a strong association, as individuals with higher education (university and postgraduate) are more likely to vape. Nationality influences vaping rates, particularly among Europeans (13.8%) compared to Southeast Asians (4.4%). Marital status is significant, with never-married individuals vaping at a rate of 8.1%, compared to 4.4% among ever-married individuals. Religion also plays a role, with Muslims reporting a lower prevalence (6.2%) compared to Christian. Employment status demonstrates a strong association, with students exhibiting the highest vaping rates (19.6%), followed by employed individuals (4.9%) and the unemployed (0.9%). However, work categories and income levels did not show significance due to low expected counts in several categories.

**Table 3:** Cross tabulation of sociodemographic characteristics and vaping behavior (*N* = 1,500).

Variable	Group	Vaping – E-cigarette user				Total	*P*
		No		Yes			
		No.	%	No.	%		
Age	18–24	363	89.9	41	10.1	404	<0.001
	25–34	498	94.9	27	5.1	525	
	35–44	238	93.7	16	6.3	254	
	45–54	185	96.9	6	3.1	191	
	>55	125	99.2	1	0.8	126	
Gender	Male	863	95	45	5	908	<0.05
	Female	546	92.2	46	7.8	592	
Education	Below University	872	98.3	15	1.7	887	<0.001
	Above University	537	87.6	76	12.4	613	
Nationality	AFR	126	98.4	2	1.6	128	<0.001
	AMR	29	90.6	3	9.4	32	
	SEAR	587	95.6	27	4.4	614	
	EUR	69	86.3	11	13.8	80	
	EMR	484	93.1	36	6.9	520	
	WPR	114	90.5	12	9.5	126	
Marital status	Never married	623	91.9	55	8.1	678	<0.01
	Ever married	786	95.6	36	4.4	822	
Religion	Muslim	948	93.8	63	6.2	1,011	<0.05
	Christian	215	91.5	20	8.5	235	
	Others	246	96.9	8	3.1	254	
Employment	Employed	890	95.1	46	4.9	936	<0.001
	Student	156	80.4	38	19.6	194	
	Self employed	130	96.3	5	3.7	135	
	Other	233	99.1	2	0.9	235	
Work categories	White collar	278	90.3	30	9.7	308	NS
	Blue collar	464	99.1	4	0.9	468	
	Green collar	17	94.4	1	5.6	18	
	Gold collar	56	88.9	7	11.1	63	
	Others	74	94.9	4	5.1	78	
Income level	Below AED 2,000	303	99.3	2	0.7	305	NS
	AED 2,000–4,999	302	97.1	9	2.9	311	
	AED 5,000–9,999	148	92.5	12	7.5	160	
	AED10,000–14,999	94	90.4	10	9.6	104	
	AED15,000–19,999	73	91.3	7	8.8	80	
	Above AED 20,000	70	89.7	8	10.3	78	

Table 4 summarizes the behavioral and psychosocial factors associated with e-cigarette use. Significant associations were observed across most variables. Individuals reporting stress related to work or studies were substantially more likely to vape (17.5%) compared to those without stress (3.6%; *p* < 0.001). Similarly, curiosity emerged as a strong motivator, with 25.6% of curious individuals using e-cigarettes versus only 2.9% among those who were not curious (*p* < 0.001). Having family members (9.5%) or friends (9.7%) who use tobacco was also strongly linked to higher vaping prevalence (*p* < 0.001). In contrast, spiritual beliefs showed no significant association with e-cigarette use. Respondents who considered the cost of smoking (25.3%) and those experiencing environmental stress (31.5%) reported notably higher vaping rates than their counterparts (*p* < 0.001). Exposure to tobacco marketing and advertising (21.9%) and peer pressure (14.6%) were additional significant correlates of vaping behavior (*p* < 0.001). Finally, individuals who reported using vaping as a coping strategy demonstrated the highest prevalence of e-cigarette use (28.8%), underscoring the strong behavioral component underlying vaping initiation and maintenance (*p* < 0.001).

**Table 4:** Factors associated with vaping – e-cigarette usage (*N* = 1,500).

Variable	Group	Vaping – E-cigarette user				Total	*P*
		No		Yes			
		No.	%	No.	%		
Stress	No	1,192	96.4	44	3.6	1,236	<0.001
	Yes	217	82.5	46	17.5	263	
Curiosity	No	1,258	97.1	38	2.9	1,296	<0.001
	Yes	151	74.4	52	25.6	203	
Family members use tobacco	No	953	95.8	42	4.2	995	<0.001
	Yes	456	90.5	48	9.5	504	
Friends use tobacco	No	631	99.1	6	0.9	637	<0.001
	Yes	778	90.3	84	9.7	862	
Spiritual beliefs	No	1,167	94.3	71	5.7	1,238	NS
	Yes	242	92.7	19	7.3	261	
Cost of smoking	No	1,335	95.4	65	4.6	1,400	<0.001
	Yes	74	74.7	25	25.3	99	
Work/Environment stress	No	1,311	96.6	46	3.4	1,357	<0.001
	Yes	98	68.5	45	31.5	143	
Marketing and advertising	No	1,359	94.7	76	5.3	1,435	<0.001
	Yes	50	78.1	14	21.9	64	
Peers/social/environments Pressure	No	1,304	94.8	72	5.2	1,376	<0.001
	Yes	105	85.4	18	14.6	123	
Coping strategy	No	1,310	96.3	50	3.7	1,360	<0.001
	Yes	99	71.2	40	28.8	139	

Crude OR: The unadjusted (crude) odds ratios revealed several significant associations with e-cigarette use among adults in the UAE. Younger adults, particularly those aged 18–24, were more likely to use e-cigarettes, with the likelihood significantly decreasing with increasing age. Females had notably higher odds of e-cigarette use compared to males (OR = 4.48, *p* < 0.001), and individuals with education above university level had substantially greater odds (OR = 11.98, *p* < 0.001). Students also had markedly increased odds of use (OR = 20.1, *p* < 0.001), as did those reporting stress, curiosity, exposure to marketing, and use of e-cigarettes as a coping strategy. Additionally, factors such as marital status, nationality (particularly European and Western Pacific regions), and Christian religion were also significantly associated with e-cigarette use in the crude analysis (Table 5).

**Table 5:** Crude Odds Ratios (ORs) for Factors Associated with E-Cigarette Use Among Adults in the UAE

**Variable**	**Group**	**Crude OR (95% CI)**	**P**	**Adjusted OR (95% CI)**	**P**
Age	18-24	1	--	1	--
	25-34	0.31(0.17-0.55)	<0.001	0.50(0.21-1.19)	NS
	35-44	0.29(0.15-0.58)	<0.001	0.46 (0.15-1.35)	NS
	45-54	0.18(0.07-0.47)	<0.001	0.85 (0.22-3.27)	NS
	>55	0.03(0.005-0.29)	<0.01	0.16 (0.01-1.63)	NS
Gender	Male	1	--	1	--
	Female	4.48(2.72-7.36)	<0.001	2.20 (1.08-4.48)	<0.05
Education	Below University	1	--	1	--
	Above University	11.98(6.55-21.89)	<0.001	11.58 (5.60-23.92)	<0.001
Nationality	AFR	1	--	Not included in final model	
	AMR	7.87(0.98-63.3)	NS		
	SEAR	2.1(0.46-9.48)	NS		
	EUR	9.62(1.82-50.88)	<0.01		
	EMR	2.80(0.62-12.5)	NS		
	WPR	7.87(1.54-40.2)	<0.05		
Marital Status	Never Married	1	--	1	--
	Ever Married	0.37(0.23-0.60)	<0.001	0.84 (0.37-1.89)	NS
Religion	Muslim	1	--	Not included in final model	
	Christian	2.09(1.13-3.85)	<0.05		
	Others	0.60(0.27-1.33)	NS		
Employment	Employed	1	--	Not included in final model	
	Student	20.1(9.09-44.42)	<0.001		
	Self Employed	0.48(0.18-1.28)	NS		
	Other	0.20(0.04-0.88)	NS		
Stress	No	1	--	1	--
	Yes	4.67(2.83-7.71)	<0.001	1.83 (0.85-3.92)	NS
Curiosity	No	1	--	1	--
	Yes	3.89(2.39-6.33)	<0.001	2.89 (1.38-6.04)	<0.01
Family Members use tobacco	No	1	--	Not included in final model	
	Yes	0.722(0.45-1.15)	NS		
Friends use tobacco	No	1	--	Not included in final model	
	Yes	1.48(0.60-3.68)	NS		
Cost of smoking	No	1	--	1	--
	Yes	4.39(2.38-8.09)	<0.001	1.03 (0.40-2.69)	NS
Work/Environment stress	No	1	--	1	--
	Yes	4.11(2.50-6.75)	<0.001	0.96 (0.37-2.48)	NS
Marketing and advertising	No	1	--	1	--
	Yes	2.94(1.41-6.14)	<0.01	1.44 (0.5-4.1)	NS
Peers/social/environments pressure	No	1	--	Not included in final model	
	Yes	1.05(0.58-1.89)	NS		
Coping Strategy	No	1	--	1	--
	Yes	6.58(3.82-11.34)	<0.001	1.77 (0.70-4.47)	NS

Adjusted OR: After adjusting significant variables for potential confounders, only a few variables remained significantly associated with e-cigarette use. Higher education continued to show a strong independent association (AOR = 11.58, *p* < 0.001), as did female gender (AOR = 2.20, *p* < 0.05), and curiosity about e-cigarettes (AOR = 2.89, *p* < 0.01). Other factors that were significant in the simple binary regression such as age, stress, marital status, employment status, and coping strategy did not show any statistical significance after adjustment, suggesting that their initial associations may have been confounded by other variables. This indicates that education level, gender, and curiosity are the most robust independent predictors of e-cigarette use among adults in this study population (Table 5).

## Discussion

In this study, the overall prevalence of ever using traditional tobacco products was 27.6%, with 14.1% identified as current smokers. The most common form of tobacco use was cigarette smoking (22.3%), followed by shisha (5.6%) and dhokha (1.4%). E-cigarette (vaping) use, which does not involve tobacco leaf, was reported by 6.1% of participants. Recent studies examining vaping prevalence among UAE university students indicate substantially higher rates compared to the general adult population. Sallam et al. (24) reported a vaping prevalence of 39.6% among university students, the highest among Arab countries analyzed.

In contrast, national estimates place vaping prevalence among UAE adults at under 1%, underscoring important subgroup differences and the influence of sampling frames (25).

Among tobacco users, 78.7% were single-product users, while 21.3% reported using more than one type of tobacco product (polyusers). Among all respondents, 3.1% were current vapers, 0.6% were former vapers, and 2.4% were classified as novice or occasional vapers.

When compared to a household study in the UAE, which indicated lower rates of current (9.1%) and former smokers (3%) (12), our findings indicate a higher prevalence of tobacco and nicotine product use. This discrepancy may stem from differences in operational definitions—our study included a broader range of products such as e-cigarettes and classified users more comprehensively (current, former, occasional). Additionally, the convenience sampling method employed may have contributed to the higher observed prevalence.

Factors influencing vaping prevalence included age, gender, education level, nationality, marital status, religion, and employment status. National health survey data from the UAE show that smoking prevalence is lowest among elders aged 60 and older and highest in the 30–44 age group (12), suggesting that smoking behaviors are more common among middle-aged adults due to longer exposure and established habits. Systematic reviews report smoking prevalence rates of 23.4–24.7% in men and only 0.8% in women (26), while a study in Abu Dhabi found tobacco use at 30% in women and 36% in men (9). Notably, postgraduate students exhibit the highest current smoking rates (17.9%), with undergraduate e-cigarette use at 16.1% (10).

Regional differences are also evident, as population data shows the highest e-cigarette usage in Europe (20.1 million) compared to Africa (5.6 million) (27). Marital status influenced both smoking and vaping behaviors: a cross-sectional study among UAE university students found a prevalence of 17.2% in married individuals versus 15% in single individuals, with single individuals showing higher rates of both e-cigarette and conventional tobacco use (10). Additionally, a national study from England highlights higher smoking rates among those with no religious affiliation (66.2% ever smoking) compared to Muslims (35.2%) (28). Employment status further affects smoking prevalence, with government employees showing the highest rates (13.7%) (26) and unemployed individuals demonstrating lower usage. Overall, these findings reflect the complex interplay of demographic and sociocultural factors influencing smoking and vaping behaviors in the UAE.

Behavioral factors also played a crucial role in vaping prevalence. While specific studies on behavioral factors influencing vaping in the UAE are lacking, international research highlights significant associations. For instance, a study conducted during the COVID-19 pandemic among Dutch smokers found that stress notably impacted smoking behavior, either increasing or decreasing it (29). Similarly, a youth risk behavior survey indicated a higher prevalence of e-cigarette use among individuals experiencing psychosocial stressors, suggesting that stress serves as a coping mechanism for many (30). Curiosity also emerges as a powerful motivator, with research among young adults in the US identifying it as a leading reason for experimenting with e-cigarettes (31). Moreover, family and peer influences significantly impact smoking behaviors; studies have shown that tobacco use is often higher among individuals from families (32) where tobacco is used, emphasizing the need for family-focused prevention strategies. Peer influence (33, 34) further reinforces these behaviors, as demonstrated in various studies that link social engagement and peer pressure to increased smoking initiation.

Environmental factors also play a role. A study among Chinese secondary school students found that active coping, school climate, and school identification were positively associated with smoking and vaping, while perceived stress was negatively associated (35), suggesting that supportive school environments may mitigate the likelihood of vaping among students. Economic factors are important as well, with studies indicating that higher tobacco prices can lead to increased cessation rates (36–38). Work-related stress has also been shown to contribute to higher smoking rates, underscoring the importance of workplace interventions (39). Exposure to tobacco advertisements has been associated with experimentation and later smoking initiation among adolescents, highlighting the substantial influence of marketing on youth smoking behaviors (40). Lastly, spiritual beliefs appear to offer a protective factor against smoking, with studies indicating lower smoking rates among individuals engaged in religious practices (28, 41).

Collectively, these findings underline the importance of addressing behavioral, economic, and psychological factors in both smoking and vaping prevention efforts and highlight the need for targeted interventions that consider these dynamics.

This study’s primary strength lies in being the first comprehensive investigation of vaping prevalence and its associated factors among adults in the UAE, thereby addressing a critical knowledge gap in the region. The large and diverse sample size (*N* = 1,500) enhances the reliability and generalizability of the findings across various demographic groups. Additionally, the study employed a rigorously validated and piloted questionnaire and utilized robust statistical methods to ensure the validity of associations identified between sociodemographic, behavioral, and environmental factors and both tobacco and e-cigarette use.

## Conclusion

This study provides valuable insights into the prevalence and patterns of both traditional tobacco smoking and e-cigarette use among adults in the UAE. The findings indicate that cigarette smoking remains the most prevalent form of tobacco use, while e-cigarette use, although lower in prevalence is an emerging behavior that warrants separate attention, Importantly, this study distinguishes between traditional tobacco use and e-cigarette use, underscoring that these behaviors are driven by different determinants.

Our findings highlight important demographic and behavioral factors influencing e-cigarette use. Non-modifiable factors such as age, gender, and nationality are significantly associated with e-cigarette use. In addition, modifiable behavioral factors—including curiosity, social influences from family and peers, and stress—play crucial roles in the initiation and continuation of e-cigarette use. The study findings support the need for targeted awareness programs among students and young adults, given the high prevalence of vaping in these groups. However, recommendations are based on observed associations rather than causal inferences, and future representative surveys are needed to confirm these patterns before large-scale policy actions. Specifically strengthening social support systems and implementing effective stress management interventions are essential to preventing initiation and promoting cessation. Such comprehensive efforts are vital to reduce the health burden posed by both traditional and emerging nicotine products in the UAE.

## Limitations

This study has several limitations. The use of convenience sampling and predominant recruitment from the Ajman emirate may limit the generalizability of the findings to the national adult population of the UAE. While random sampling is often challenging to implement in community-based surveys of this nature, the overrepresentation of blue-collar workers further restricts population representativeness. The cross-sectional design precludes causal inference, and reliance on self-reported data introduces potential recall and social desirability biases impacting the accuracy of tobacco and e-cigarette use reporting. Nonetheless, the primary aim was to determine the prevalence of e-cigarette use and explore associated factors within the study population, focusing on quantifying the magnitude of the issue rather than developing or testing behavioral theories. Future research could build on these findings by applying established behavioral models such as the Health Belief Model or Theory of Planned Behavior. Despite these constraints, the study offers valuable insights to inform public health policies and guide future tobacco and nicotine product research in the UAE.

## Data Availability

The raw data supporting the conclusions of this article will be made available by the authors, without undue reservation.

## Supplementary material

SUPPLEMENTARY FILE S1 English version of the questionnaire.
