# Supplementary material for: Prevalence and patterns of traditional and e-cigarette use, and factors associated with e-cigarette use
Source: Front Public Health. 2025 Dec 18;13:1698254. doi: 10.3389/fpubh.2025.1698254 (PMC12756352; doi:10.3389/fpubh.2025.1698254)
Supplement: SUPPLEMENTARY FILE S1 — English version of the questionnaire. [file Data_Sheet_1.PDF]

Gulf Medical University, Ajman, UAE

Questionnaire

Vaping Patterns Among Adults in the United Arab Emirates: Novices, Current Users, Ex-Smokers, and Quitting Efforts

Section 1

Socio-demographic Characteristics

Age *

Above 18 years only 0/2

Gender *

- ◯ Male
- ◯ Female

Education *

- ◯ Did not attend or complete primary school
- ◯ Primary School
- ◯ Middle School
- ◯ Secondary School
- ◯ University
- ◯ Postgraduate (eg Masters or PhD)

Nationality *

Please Select

Marital Status *

- ◯ Single
- ◯ Married
- ◯ Divorced
- ◯ Widowed
- ◯ Separated

What is your current religion, if any? *

- Muslim
- Christian
- Hindu
- Buddhist
- Sikhism
- Jewish
- Judaism
- Other

Emirate/ City of residence: *

Please Select

Employment Status: *

- Employed
- Unemployed
- Student
- Self Employed
- Retired

If employed, what type of work *

- White Collar (Salaried Professionals)
- Blue Collar (Working class, manual labour or skilled trades)
- Pink Collar (Working class, service industry)
- Red Collar (All kind of Civil Servants)
- Green Collar (Conservation and sustainable sector)
- Gold Collar (Specialized field of law and medicine)
- Other

Income Level (in AED): *

- < 2,000
- 2,000 - 4,999
- 5,000 -9,999
- 10,000 – 14,999
- 15,000 – 19,999
- 20,000 – 29,999
- ≥30,000
- Prefer not to answer

Section 2

Factors related to smoking & Smoking History

For each of the following statements, please indicate if it applies to you by answering 'Yes' or 'No'. *

	Yes	No
Are you stressed with your work/studies?	◯	◯
Curiosity is one of the reasons for starting smoking.	◯	◯
Do any of your family members use tobacco?	◯	◯
Do any of your friends use tobacco?	◯	◯
Do spiritual beliefs guide your decisions regarding smoking?	◯	◯
Does the cost of smoking influence your smoking behaviour?	◯	◯
Will the work environment or living environment stress influence your smoking behaviour?	◯	◯
Has tobacco marketing and advertising influenced your smoking behaviour?	◯	◯
Have you experienced pressure from peers or social environments to smoke?	◯	◯
Is smoking used as a coping strategy for stress or emotional management?	◯	◯

How do you perceive the health risks associated with smoking? *

◯ Very Concerned
◯ Somewhat concerned
◯ Not concerned at all

Have you ever smoked tobacco? *

◯ Yes
◯ No

Have you smoked more than 100 cigarettes(5 packs of 20 cigarettes or 4 packs of 25 cigarettes) / 20 sessions of vaping (e-cigarette) or 20 of any other tobacco in your entire life? *

- ◯ Yes
- ◯ No

Which type of tobacco: *

- ☐ Cigarette
- ☐ Vaping - E Cigarette
- ☐ Shisha
- ☐ Dhokha/Midwakh
- ☐ Other

What factors do you think influenced your decision to start smoking ? Please select all that apply *

- ☐ Accessibility
- ☐ Affordability
- ☐ Exposure to e cigarette
- ☐ Family influence
- ☐ Media influence
- ☐ Coping strategy
- ☐ Curiosity about the taste or experience
- ☐ Experimental
- ☐ Friend Influence
- ☐ Other

Have you been smoking tobacco since the past 12 months? *

- ◯ Yes
- ◯ No

If yes, pattern on use of tobacco smoking - Number of Cigarettes per day *

If yes, pattern on use of tobacco smoking - Number of Shisha per day *

If yes, pattern on use of tobacco smoking - Number of Dhokha/ Midwakh per day *

If yes, pattern on use of tobacco smoking - Number of E-cigarette (Vape) per day *

Please tick on the duration for each tobacco use: *

	< 6 months	6 mon-1 yr	>1 Year	Not Applicable
Cigarette	○	○	○	○
Dokha/Midwakh	○	○	○	○
Shisha	○	○	○	○
Vaping (E-cigarette)	○	○	○	○

Section 3

E-cigarette Use, please provide the following details

Are you an E-cigarette - past/current (vaping) user? *

- ◯ Yes
- ◯ No

Age at start of E-cigarette: *

0/3

Reason for starting E-cigarettes? (Select all that apply) *

- ☐ To quit traditional tobacco smoking
- ☐ To reduce the harm of traditional tobacco smoking
- ☐ Curiosity
- ☐ Peer pressure
- ☐ Flavor/taste
- ☐ No smell
- ☐ Prestige
- ☐ Other

What type of vaping device do you primarily use? *

- ◯ Cig-a-Like
- ◯ Vape pen
- ◯ Box mod
- ◯ Pod system
- ◯ Other

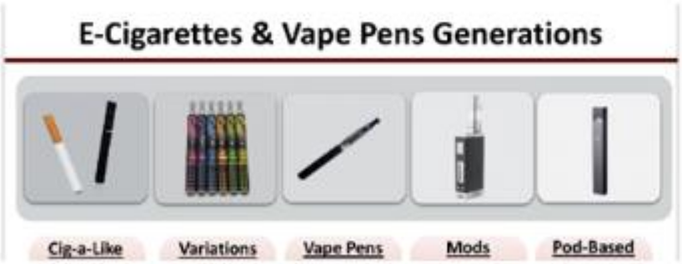

How did you first hear about e-cigarettes? *

- ◯ Online advertising
- ◯ Social media
- ◯ Friends or family
- ◯ Other

What flavors of e-cigarettes do you prefer? (Select all that apply) *

- ☐ Fruity
- ☐ Minty
- ☐ Tobacco
- ☐ Dessert
- ☐ Other

How important are flavors in your decision to use e-cigarettes *

- ◯ Very important
- ◯ Somewhat important
- ◯ Not important

Do you use e-cigarettes as a substitute for traditional cigarettes or as an addition to smoking? *

- ◯ Substitute
- ◯ Addition
- ◯ Both

Following are statement related to quitting and vape *

	Yes	No
Have you ever attempted to quit vaping?	◯	◯
Have you experienced any withdrawal symptoms when trying to quit e-cigarettes?	◯	◯

Age at which you quit e-cigarette (If past user) *

____

If not a user mention 0

Section 4

Factors Driving E-cigarette Use

Where do you buy your vape most of the time? *

- ◯ Vape shop
- ◯ Mall
- ◯ Friend or relative
- ◯ Online
- ◯ Other

What do you perceive/believe to be as the advantages of using E-cigarettes over traditional cigarettes? (Select all that apply) *

- ☐ Less harmful to health
- ☐ Easier to quit smoking
- ☐ Availability of flavors
- ☐ Social acceptance
- ☐ Other

Are there any concerns or disadvantages you associate with E-cigarette use? (Select all that apply) *

- ☐ Health risks
- ☐ E-cigarette addiction
- ☐ Lack of regulation
- ☐ Social stigma
- ☐ Other

Section 5

Quitting efforts

Have you ever made an attempt to quit smoking? *

- ◯ Yes, I successfully quit after one attempt.
- ◯ Yes, but I needed multiple attempts before successfully quitting.
- ◯ Yes, I have attempted to quit but am currently still smoking.
- ◯ No, I have not attempted to quit smoking.
- ◯ Not applicable/I am not a smoker.

Have you stopped smoking for at least 1 day because you were trying to quit smoking? *

- ◯ Yes
- ◯ No

How successful do you feel your past quitting efforts were? *

1 2 3 4 5

Not at all — Extremely

How interested are/were you in quitting smoking? *

1 2 3 4 5

Not at all — Extremely

What factors do you think would influence or influenced your decision to stop smoking? Please select all that apply *

- ☐ Accessibility
- ☐ Affordability
- ☐ Exposure to e cigarette
- ☐ Family influence
- ☐ Media influence
- ☐ Coping strategy
- ☐ Health risk
- ☐ Religion
- ☐ Other

If you are an Ex-user or had a quit attempt of traditional tobacco, please provide details on method used to quit (Select all that apply) *

- [ ] Nicotine replacement
- [ ] Electronic Cigarette
- [ ] Nicotine gum
- [ ] Smoking cessation counselling
- [ ] Get support to stop
- [ ] Will power

Thank you for participating in this survey! Your input is valuable for our research.
